# Complete chloroplast genome sequence of *Lens ervoides* and comparison to *Lens culinaris*

**DOI:** 10.1038/s41598-022-17877-7

**Published:** 2022-09-05

**Authors:** Nurbanu Tayşi, Yasin Kaymaz, Duygu Ateş, Hatice Sari, Cengiz Toker, M. Bahattin Tanyolaç

**Affiliations:** 1 Bioengineering Department, Faculty of Engineering, Ege University, Izmir, Turkey; 2 Department of Field Crops, Faculty of Agriculture, Akdeniz University, Antalya, Turkey

**Keywords:** Agricultural genetics, Plant sciences

## Abstract

*Lens* is a member of the Papilionoideae subfamily of Fabaceae and is generally used as a source of vegetable protein as part of human diets in many regions worldwide. Chloroplast (cp) genomes are highly active genetic components of plants and can be utilized as molecular markers for various purposes. As one of the wild lentil species, the *Lens ervoides* cp genome has been sequenced for the first time in this study using next-generation sequencing. The de novo assembly of the cp genome resulted in a single 122,722 bp sequence as two separate coexisting structural haplotypes with similar lengths. Results indicated that the cp genome of *L. ervoides* belongs to the inverted repeat lacking clade. Several noteworthy divergences within the coding regions were observed in *ndh*B, *ndh*F, *rbc*L, *rpo*C2, and *ycf*2 genes. Analysis of relative synonymous codon usage showed that certain genes, *psb*N, *psa*I, *psb*I, *psb*E, *psb*K, *pet*D, and *ndh*C, preferred using biased codons more often and therefore might have elevated expression and translation efficiencies. Overall, this study exhibited the divergence level between the wild-type and cultured lentil cp genomes and pointed to certain regions that can be utilized as distinction markers for various goals.

## Introduction

Lentil (*Lens culinaris* subsp. *culinaris*) is an herbaceous, self-pollinating, annual legume species, one of the first domesticated plants by humans^1^. The genus *Lens* is a member of the Papilionoideae subfamily in the Fabaceae. Cultivated lentils and wild species are widespread in Southwest Asia and the Mediterranean region^2^. Lentil, a cool-season plant, is generally used as a vegetable protein source as part of human diets in many parts of the world^3^. It contributes to overcoming malnutrition as a staple food with its high protein content, especially for children in low-income countries with substantial protein deficiencies^4^. It also benefits the local environment by fixing nitrogen in root nodules and establishing a symbiotic relationship with *Rhizobium*, thus improving soil fertility^4,5^. At the same time, its wild species are useful resources for adaptive traits, such as disease resistance, abiotic stress tolerance, and pesticide resistance^6^. *Lens ervoides* is one of the wild species that belongs to the third of the four gene pools with seven species^7^, and its natural habitat surrounds Turkey, Israel, Syria, the Adriatic Coast, Southern Italy, subregions of Spain, and Algeria^1^.

The *L. culinaris* genome has a diploid structure (2n = 14), and its chloroplast (cp) genome is 122,967 bp^8^. Relative to the nuclear genome, the cp has a slower evolutionary rate due to fewer nucleotide substitutions and recombination events^9^. The cp is present in plants in their photosynthetically active green tissues and exhibits an essential role as an organelle^10^. Additionally, the cp plays a crucial role in biochemical processes, photosynthesis, protein-coding, and the arrangement of secondary metabolic activities^11,12^. The cp has its genetic material separate from the nucleus and its genome, generally 120 to 170 kb in size, and encodes for 120 to 130 genes^13^. The cp genome usually has a quadripartite structure with a large single copy, a small single copy, and two inverted repeat (IRa and IRb) regions^14^. Homologous recombination occurs between these two inverted regions^15,16^. This phenomenon, called flip-flop recombination, allows an individual plant to have coexisting alternative structural haplotypes^17,18^. Although the cp genome structure is mostly conserved, an expansion of the IR regions has been observed among various species, whereas one of the IR copies has been lost in Fabaceae plants^19^. The conserved structure of the cp genomes due to their slow evolution and maternal inheritance answers important questions of plant phylogenetics through comparative analyses on closely related species^20^. Each cell contains ~ 100 cp, and their large copy number genome is advantageous for cp genetic engineering^21^. Therefore, cp genome sequences are highly attractive in evolutionary phylogenetic analyses, agricultural biotechnology, and barcoding studies.

Wild plant species can carry strong features against harsh environmental conditions. Such properties can enrich current agricultural practices; therefore, associated genotypes should be investigated. However, a high-quality cp genome of a wild lentil species has yet to be sequenced. Low-cost high-throughput next-generation sequencing (NGS) technologies have been extensively utilized in plant genomics^22,23^. The cp genomes of various species are being sequenced rapidly and shared through public domains^24^. Therefore, this study tackled the challenge of sequencing the sole cp genome of a wild lentil species.

This study sequenced the complete cp genome of *L. ervoides* for the first time. The results were compared to the previously sequenced complete *L. culinaris* cp genome. This study aimed to determine how the *L. ervoides* cp genome is structurally organized in terms of gene content and its divergence relative cp genome of *L. culinaris*. This study identified obvious cp sequence differences that can be used to develop markers and elucidated a phylogenetic relationship among relative species.

## Results

### Wild lentil, *L. ervoides*, cp contains two separate genomes with different structural orientations

**Figure 1:**
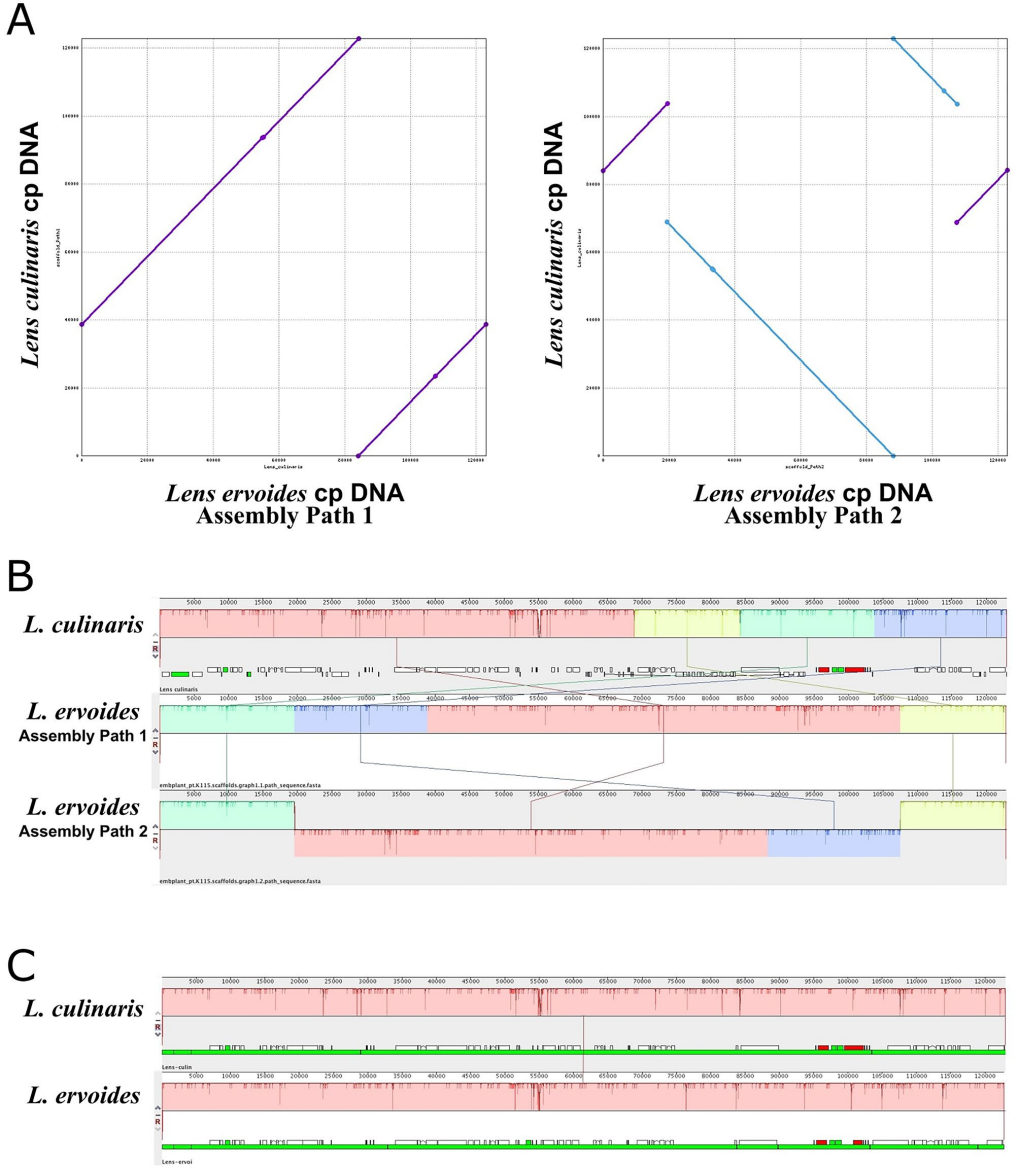
(**A**) Comparison of two possible *L. ervoides* cp genome scaffold orientations (Path 1—left panel; Path 2—right panel) to *L. culinaris* cp genome sequence using nucmer. Mummerplot analysis demonstrates similar sequence blocks with the same orientation as purple diagonal lines while inverted reverse sequence blocks as backward diagonal lines in blue colour. (**B**) Gene order comparison of *Lens* cp genomes using MAUVE software. Blocks of the same colour show the same gene orientation. Boxes above the line represent the clockwise gene sequence, and boxes below the line represent gene sequences in the opposite direction. Co-existing alternative structural haplotypes (scaffolds graph 1 and 2) of *L. ervoides* and comparison of these structures with *L. culinaris*. (**C**) Block similarity alignments of both genomes after manually setting the starting point of *L. ervoides* assembly path 1 according to *L. culinaris*.

The cp genome of *L. ervoides* was sequenced using the BGISEQ-500 platform. The de novo cp genome was created with a widely used pipeline called GetOrganelle, specifically designed for organelle array structures. After obtaining the final assemblies, the average depth of coverage was calculated as 6800X. Of the 14,281,896 raw reads (150 bp paired-end reads), 2,794,255 read pairs (19.5%) were derived from the cp genome of *L. ervoides*. The de novo genome assembly resulted in two separate scaffolds (assembly paths 1 and 2). Therefore, these assemblies were investigated with a comparative analysis against the previously sequenced *L. culinaris* cp genome using nucmer. The first scaffold (assembly path 1) followed the same structural orientation as *L. culinaris*, although the starting point occurred at a different location due to the stochasticity of the de novo assembly process (Fig. 1A, left). This was expected given that the cp genomes are naturally circular. However, the secondary scaffold (assembly path 2) contained a partially inverted orientation as opposed to *L. culinaris* cp, which appears in two large blocks next to each other (Fig. 1A, right). These two scaffolds are not complementary as they form independently complete genomes without missing any piece. These structural haplotypes were further validated by a separate analysis, and similarity blocks were obtained against reference *L. culinaris*. Mauve analysis showed similar results to nucmer analysis, further supporting the orientation differences between the assembled scaffolds (Fig. 1B). Because these two structural variants were also analogous to each other in terms of sequence compositions other than regional inversions and a slide difference in length (122,722 vs. 122,834 bp), the first assembly with the same orientation as the reference was manually modified to have the same starting point and used for the downstream analysis (Fig. 1C).

### *L. ervoides* cp genome is profoundly similar to *L. culinaris*

**Figure 2:**
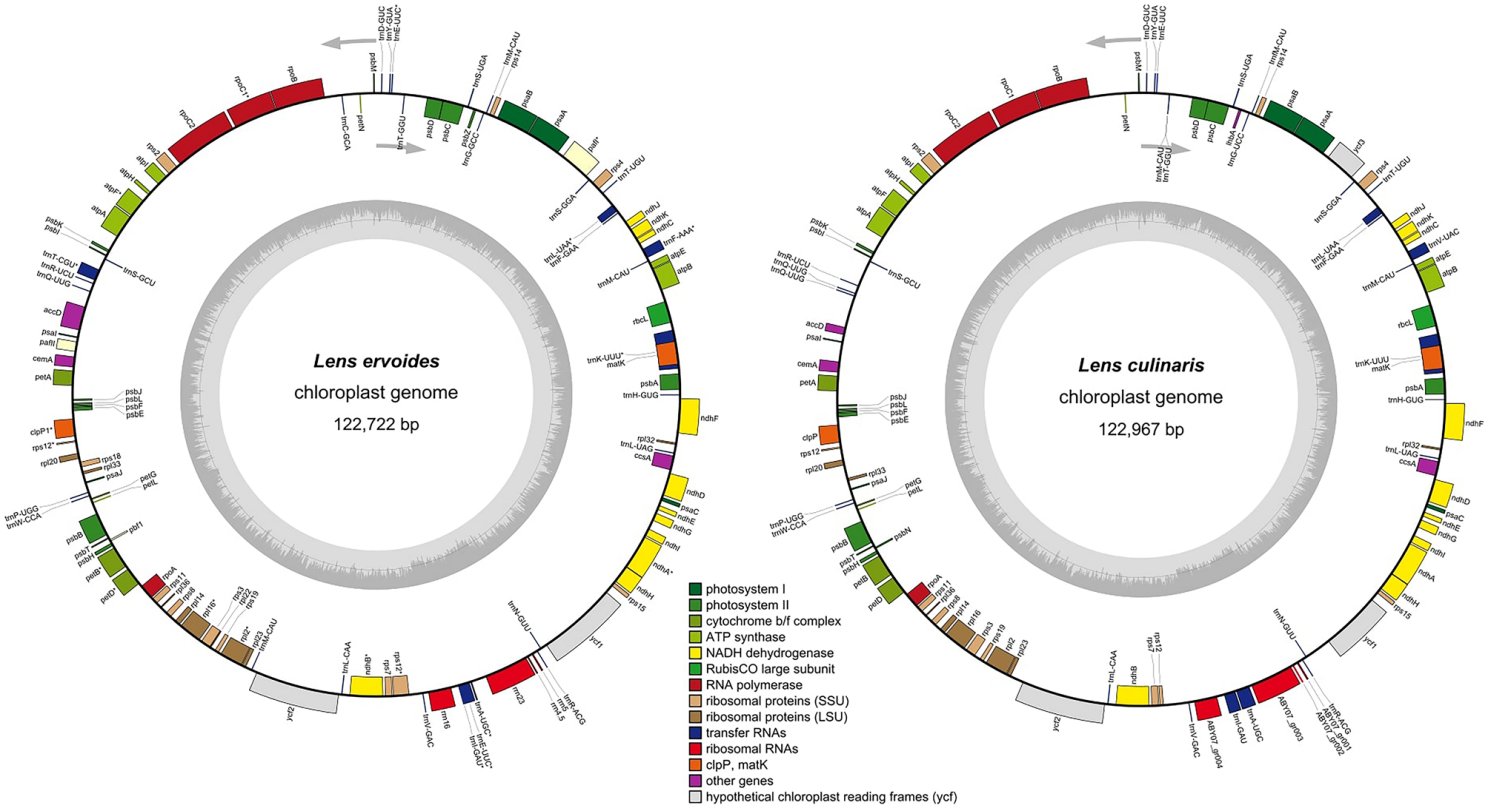
Gene maps of *L. ervoides* and *L. culinaris*. Genes inside the circle transcript clockwise, and genes outside the circle transcript counterclockwise. Genes with the same functional trait are shown in the same colour group. The dark grey area in the inner circle indicates the GC content of the corresponding genes, and the light grey area indicates the AT content.

As an initial examination, the gene content of *L. ervoides* was determined and compared to *L. culinaris*. Their genome lengths were 122,722 and 122,967 bp, respectively (Table 1). The *L. ervoides* cp genome is circular and lacks the typical quadripartite cp genome structure and therefore belongs to the family of inverted repeat lacking clade (IRLC). Although *L. ervoides* and *L. culinaris* were structurally quite the same and no large regions were missing between the two, this gene annotation analysis resulted in disagreements in terms of the presence of certain genes. In particular, *rp*l22, *rps*18, *ycf*4 (*paf*II), and one *trn*M-CAU gene were missing, and *ycf*1 appeared as a pseudogene in *L. culinaris*. Another disagreement in *L. culinaris* was observed between tRNA genes. Some tRNA genes were altered, whereas the *trn*M-CAU gene was missing. The reason was the use of an obsolete annotation tool, DOGMA, for the *L. culinaris* genome deposited to GenBank. All genes, except *trn*T-CGU, were covered and matched once; the same annotation method, namely GeSeq, was used. OGDRAW visualization of both cp genomes and the gene orientations is shown in Fig. 2.

**Table 1:** Comparison of *L. ervoides* cp genome and *L. culinaris* cp genome deposited to the GenBank features.

Species	*L. ervoides*	*L. culinaris*
Chloroplast genome size (bp)	122,722	122,967
Total number of genes	112	107
Protein coding genes	77	73
tRNA genes	31	29
rRNA genes	4	4
Pseudogene	–	1
Overall GC content (%)	34.44	34.4

The number of genes for *L. ervoides* and *L. culinaris* was determined as 112 and 107, respectively. Table 2 summarizes the genes encoded by *L. ervoides* based on gene functions. Among protein-coding genes, *rpl*2, *ndh*A*, ndh*B, *rp*oC1, *atp*F, and *clp*P contain two exons, whereas *ycf*3 (*paf*I) has three exons. The *rps*16 gene was lost in all legumes^25^. This analysis confirmed the lack of the *rps*16 gene in either species. Additionally, an intronic region loss of the *clp*P gene, which has been previously reported for the *L. culinaris* cp^26^, has also been observed in the *L. ervoides* cp genome.

**Table 2:** List of chloroplast genomes gene content and functional classification in *L. ervoides*.

Category	Functional groups	Genes
Self-replication	Large subunit of ribosomal proteins	*rpl*2^(a,c)^*, rpl*14*, rpl*16*, rpl*2*0, rpl*22 *,rpl*23*, rpl*32*, rpl*33*, rpl*3*6*
	Small subunit of ribosomal proteins	*rps*2*, rps*3*, rps*4*, rps*7*, rps*8*, rps*11*, rps1*2*, rps*14*, rps*15*, rps*19*, rps1*8
	DNA-dependent RNA polymerase	*rpo*A*, rpo*B*, rpo*C1^a,c^*, rpo*C2
	Ribosomal RNA genes	*rrn*4*.5, rrn*5*, rrn*16*, rrn*23
	Transfer RNA genes	*trn*A-UGC, *trn*C-GCA, *trn*D-GUC, *trn*E-UUC, *trn*F-AAA, *trn*F-GAA, *trn*G-GCC,
		*trnH-GUG, trnI-GAU,trn*K-UUU, *trn*L-CAA, *trn*L-UAA, *trn*L-UAG, *trn*M-CAU,
		*trn*S-GCU, *trn*S-GGA, *trn*S-UGA, *trn*T-CGU, *trn*T-GGU, *trn*T-UGU, *trn*V-GAC,
		*trn*Y-GUA, *trnQ*-UUG, *trnW*-CCA
Genes for photosynthesis	Photosystem I	*psa*A*, psa*B*, psa*C*, psa*I*, psa*J
	Photosystem II	*psb*A*, psb*B*, psb*C*, psb*D*, psb*E*, psb*F*, psb*H*, psb*I*, psb*J*, psb*K*, psb*L*,*
		*psbM, psbN *(*pbf1*)*,psbT,psbZ *(*ıhbA*)
	RUBISCO	*rbc*L
	Subunits of ATP synthase	*atp*A*, atp*B*, atp*E*, atp*F^a,c^*, atp*H*, atp*I
	Subunit of NADH-dehydrogenase	*ndh*A^a,c^*, ndh*B^a,c^*, ndh*C*, ndh*D*, ndh*E*, ndh*F*, ndh*G*, ndh*H*, ndh*I*, ndh*J*, ndh*K
	Cytochrome b/f complex	*pet*A*, pet*B*, pet*D*, pet*G*, pet*L*, pet*N
Other genes	Protease	*clp*P^a,c^
	Maturase	*mat*K
	Envelope membrane protein	*cem*A
	Translation initiation factor	*–*
	C-type cytochrome synthesis gene	*ccs*A
	Subunit of Acetyl-CoA-carboxylase	*accD*
Genes of unknown function	Conserved hypothetical chloroplast	*ycf1, ycf2, ycf3*(*pafI*)^b,d^*, ycf4*(*pafII*)

^a^Gene containing one intron.

^b^Gene containing two introns.

^c^Gene with two exons.

^d^Gene with three exons.

### Divergent hotspots mostly accumulate within intergenic regions

**Figure 3:**
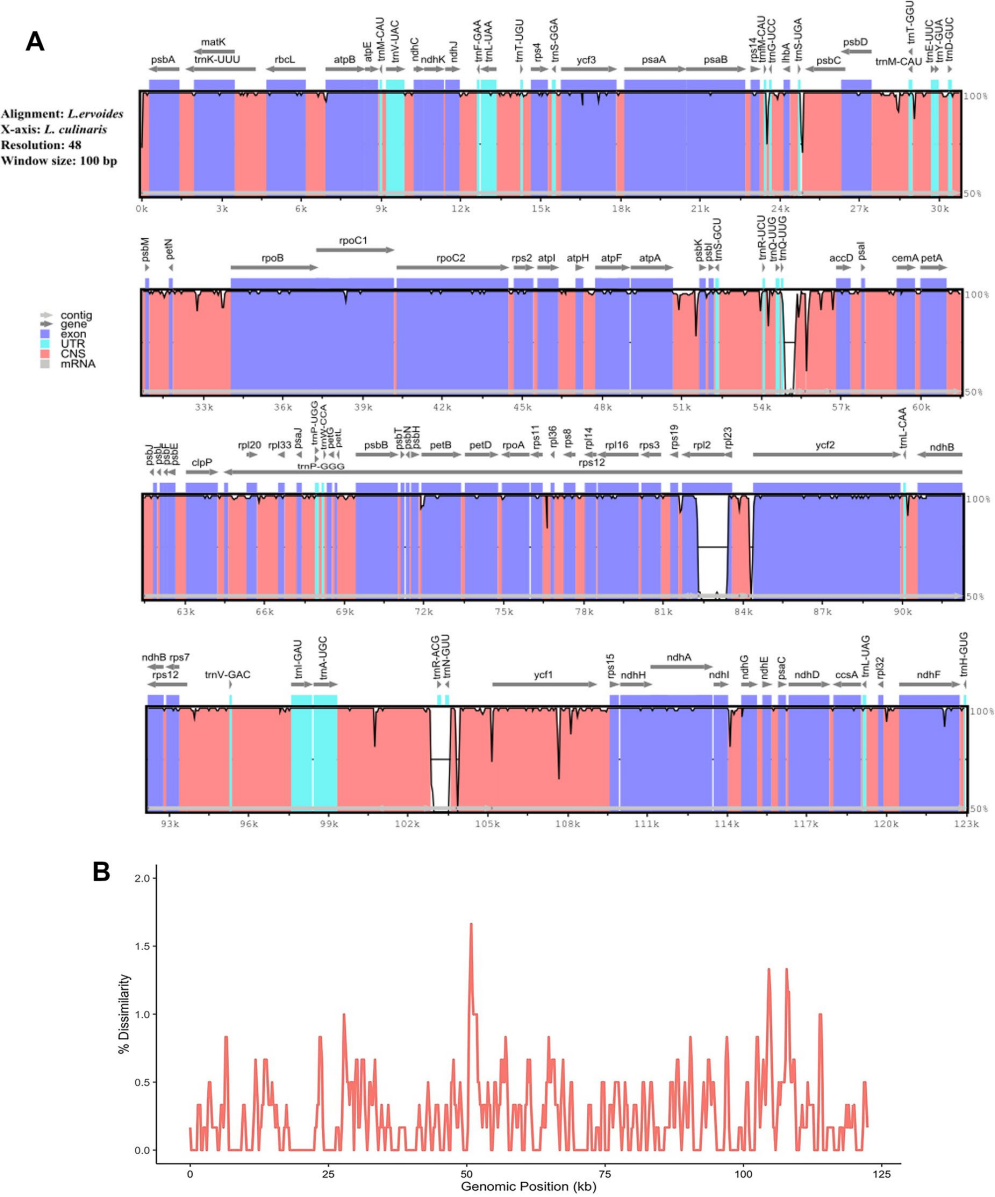
(**A**) mVISTA analysis of two cp genomes. Grey arrows show the direction of gene translation. The y-axis gives percent identity ranging from 50–100% and exon, conserved noncoding sequence (CNS), tRNAs, tRNAs are represented in different colours. (**B**) Percent dissimilarity (mismatches) between *L. ervoides* and *L. culinaris* throughout their cp genomes with 1 kb window and 200 bp sliding step. Repetitive regions determined by miropeats are masked.

The focus has been on loci that are divergent between the cp genomes of two lentil species. Given that the gene compositions were extensively similar, sequence variations associated with driving the species separation were examined. mVISTA analysis that compared the *L. culinaris* and *L. ervoides* cp sequences as windows along the genome (window size 100 bp, resolution 48) showed that most sequence disagreements accumulated within the intergenic regions, with a few exceptions of noncoding genes, such as *trn*R-ACG and *trn*N-GUU (Fig. 3A). All protein-coding genes demonstrated a high conservation level based on the global view of similarities. To prevent false divergence due to artificial alignments of repetitive sequences especially given the side-effect of assembly with short reads, the genomes were examined with a repetitive sequence detection tool, miropeats (version 2.02). miropeats determined 11 repetitive regions between 108 and 224 bp on the *L. culinaris* cp genome (Supplemental Table 1), masked during the comparative divergence analysis. After masking such regions, 305 single variations were detected between the two cp genomes, which reflect an overall ~ 0.25% dissimilarity along the genomes (Fig. 3B). The genomic regions as the divergence hotspots reaching > 1.0% sequence divergence were observed between 50,600 and 51,200, 104,600 and 105,000, and 107,800 and 108,400 bp, which also fell into intergenic regions.

### Nucleotide variations within coding and noncoding genes

Single nucleotide variations (including indels) that might affect the transcription sequence of genes have been further investigated. The possible amino acid changes due to nucleotide substitutions (nonsynonymous in coding genes) or synonymous changes were determined. For this, the coding sequence and gene coordinates from the reference *L. culinaris* were used. Based on the analysis, 30 of 73 protein-coding genes that carried at least one nucleotide substitution were detected (Supplemental Table 2). Of these 30 proteins, 18 contained at least one nonsynonymous change that resulted in a different amino acid. Among these, *ndh*B, *ndh*F, *rb*cL, *rpo*C2, and *ycf*2 showed more than three amino acid changes in *L. ervoides* cp compared to *L. culinaris*. *ndh*F also had a deletion resulting in a loss of four amino acids in-frame at position 560 (Supplemental Fig. 1A). Besides *ndh*F and *ycf*2, protein-coding genes can be considered highly conserved to a great extent. *ndh*F encodes for a protein that takes a role as an oxidoreductase subunit, whereas *ycf*2 is a hypothetical protein with an unclear function.

After expanding the examination to the sequence variation analysis for the noncoding genic regions, of 32 noncoding regions, only tRNA coding gene *trnQ*-UUG and *rrn*23 that codes for 23S rRNA carried sequence variations in the *L. ervoides* cp genome compared to their counterpart in *L. culinaris* (Supplemental Table 3). The 23S rRNA transcript carried five variants, including the insertion of 31 nucleotides (Supplemental Fig. 1B).

### Characterization of repeat sequences and simple sequence repeats (SSRs)

**Figure 4:**
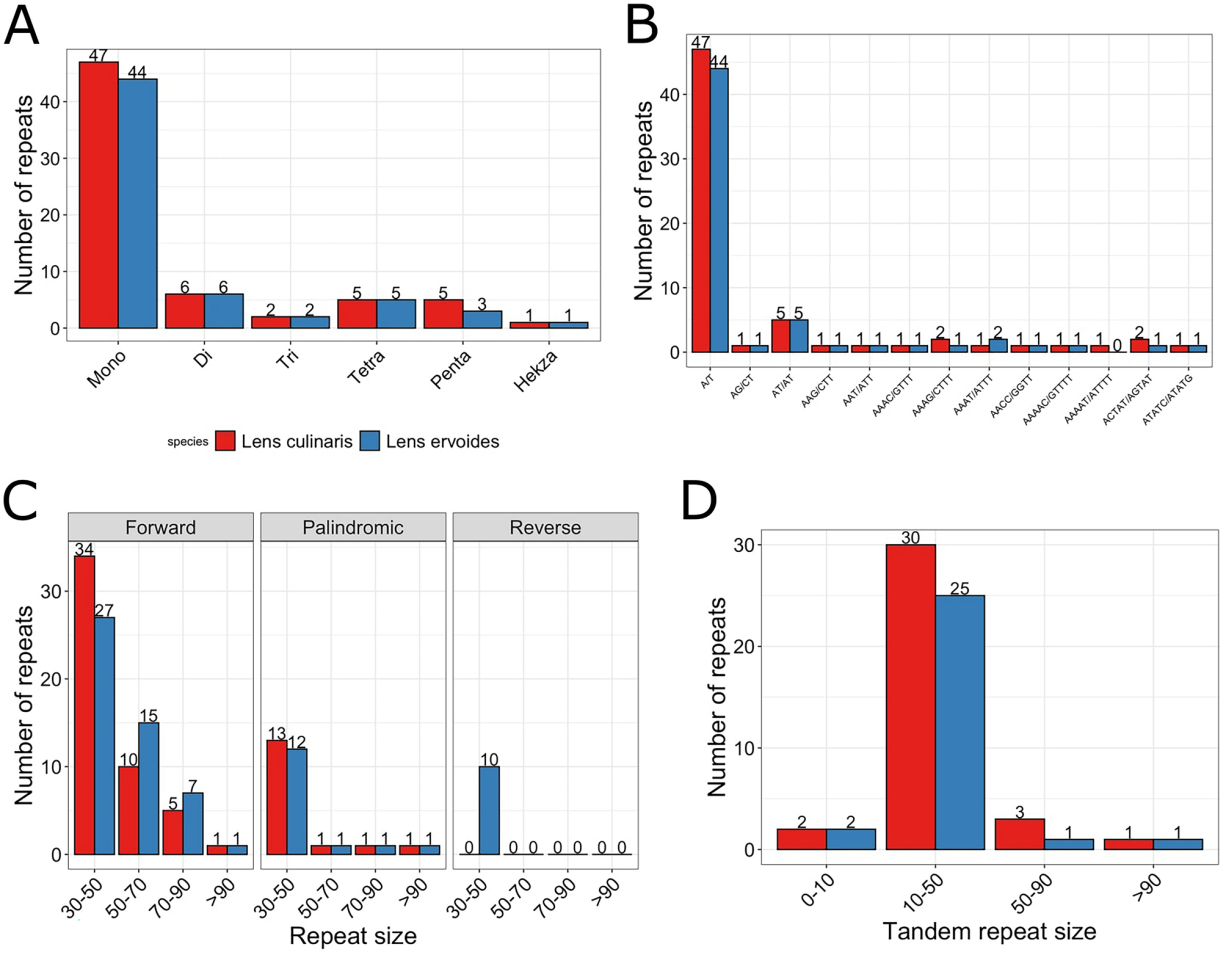
Type of simple sequence repeats (SSRs) and repeat sequences number in the cp genome of *L. culinaris* (red) and *L. ervoides* (blue). (**A**) Distribution of various SSRs on both cp genomes grouped based on type. (**B**) The frequencies of SSR types. (**C**) Various types of repeats grouped based on their size in bp. (**D**) The distribution of tandem repeats.

SSRs are commonly found in various species and consist of nucleotide sequences of one to six repeats^27,28^. The number of SSRs in *L. culinaris* (66 in total) was higher than in *L. ervoides* (61 in total; Fig. 4A). SSR types of A/T were more abundant than G/C in the cp genome of two *Lens* species (Fig. 4B), and the presence of C/G repeat types was rare^29^.

The cp genomes of both *L. culinaris* and *L. ervoides* were found to have equal numbers of forward repeats (n = 50) as the most abundant type, although a subset of them varied in size (Fig. 4C). Both genomes showed equivalent numbers of forward repeats, and *L. culinaris* carried no reverse repeats as opposed to *L. ervoides*, with 10 of them in size ranging between 30 and 50 bp. Regarding tandem repeats found in both genomes, the frequencies looked roughly similar, with slightly more repeats in the 10 to 50 bp repeat class for *L. culinaris* and *L. ervoides* (a total of 36 and 29 repeats), respectively (Fig. 4D).

### Analysis of codon usage frequencies

**Figure 5:**
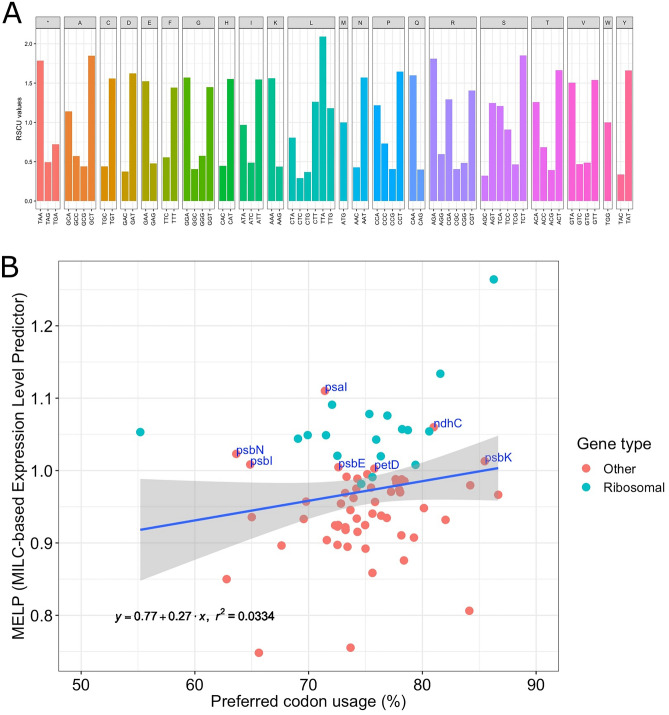
(**A**) RSCU values of each codon grouped based on their associated amino acid (single letters on top). (**B**) The relationship between the percent preferred codon (RSCU > 1.0) usage of each protein coding gene and their MELP index. Ribosomal genes are in green and other genes are read. Only non-ribosomal genes with MELP > 1.0 are labelled with gene names. Blue line represents the linear regression and the grey area surrounding the line shows the confidence interval.

The codon usage patterns of 73 protein-coding genes were investigated to examine synonymous codon preferences. These genes encoded for 19,585 codons throughout the *L. ervoides* cp genome (~ 50% of the genome). The normalized frequency of each codon type in relative synonymous codon usage (RSCU) showed the preferred synonymous codon of each amino acid (Fig. 5A). RSCU represents the ratio of observed codon frequency among all coding sequences to the expected uniform usage. RSCU values > 1.0 suggest a strong preference for the codon, whereas smaller values represent underuse. Each of the 18 amino acids, except two single-codon amino acids, methionine (M) and tryptophan (W), had one codon with an RSCU value > 1.5, indicating strongly preferred codons. Among these, the TTA codon for leucine had an RSCU value of 2.09, suggesting a highly frequent usage along the genome. Leucine was also the most abundantly encoded amino acid in the *L. ervoides* cp genome, with a total of 2108 amino acids (Supplemental Table 4).

Quantitative codon usage analysis can lead to in silico prediction of gene expression status. Predicted expression [Measure Independent of Length and Composition (MILC)-based Expression Level Predictor (MELP) value] based on relative frequencies of codon usage (MILC value; see “Materials and methods” for details) were investigated for each protein-coding gene in the *L. ervoides* cp genome using ribosomal protein-coding genes as the reference group. The MELP value for a gene is calculated as the ratio of its MILC distance to an average codon usage observed from the entire genome to its MILC distance to the reference (supposedly highly expressed) genes (ribosomal genes in this case)^30^. Higher MELP values predict high expressivity as the indication of codon usage bias. Overall, gene preferred codon usage (codons with RSCU > 1.0) rates demonstrated a suggestive positive correlation with the gene predicted expressivity (linear regression model, *r*^2^ = 0.0334; Fig. 5B). The analysis of coding genes in the *L. ervoides* cp resulted in several nonribosomal genes with MELP values > 1.0 along with ribosomal protein-coding genes. Among these genes, *psb*N, *psa*I, *psb*I, *psb*E, and *psb*K function in photosystem I/II, whereas *pet*D is in cytochrome complex and *ndh*C is an NADH-dehydrogenase subunit.

### Phylogenetic analysis

**Figure 6:**
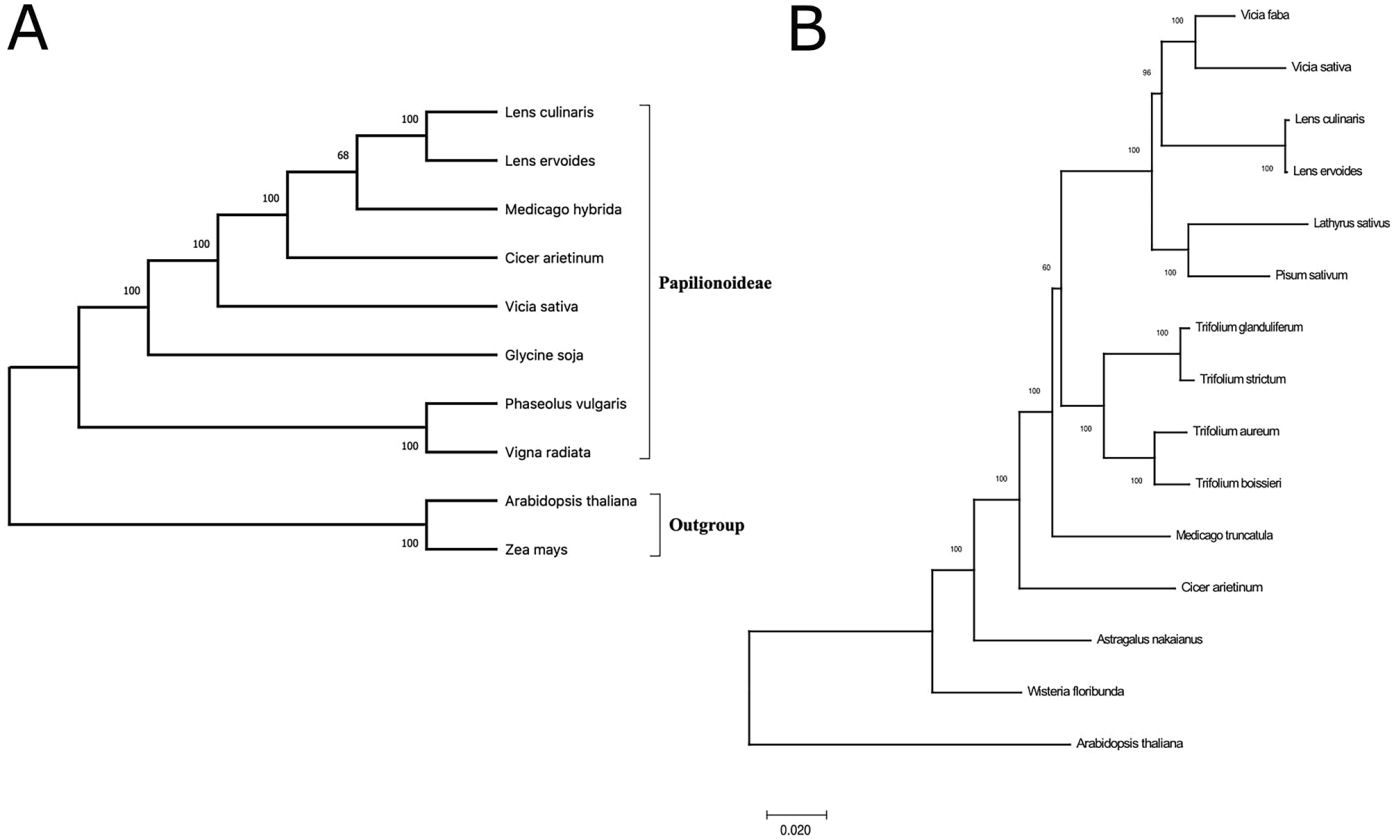
Phylogenetic relationship of Papilionoideae species using maximum likelihood (ML) and Jukes Cantor model analysis. (**A**) Phylogenetic tree of whole cp genomes from various species including IRLC and two outgroups. (**B**) Phylogenetic tree based on 54 protein coding genes that are common among 14 Papilionoideae cp genomes within IRLC. *Arabidopsis thaliana* is the out group. Bootstrap supports were calculated from 100 replicates.

Given the highly conserved nature of cp genomes among *Lens* species, the phylogenetic relationship was investigated more deeply between *L. ervoides* and the several other members of Papilionoideae. The complete cp genomes from six different species and two group representatives, *Zea mays* and *Arabidopsis thaliana*, were included in the phylogenetic tree structure (Fig. 6A). Both *Lens* genomes were clustered the closest with *Medicago hybrida* and then with *Cicer arietinum* in this analysis, whereas *Phaseolus vulgaris* and *Vigna radiata* formed their clique separate from the rest. This complete genome-based phylogenetic analysis demonstrated a noteworthy variation even within Papilionoideae.

Interspecies cp diversity was further elaborated by including more Papilionoideae species and focusing on common protein-coding genes. For this purpose, 54 protein-coding genes shared by all 14 cp genomes from Papilionoideae members of IRLC were examined, and a phylogenetic tree on their protein sequences was constructed (Fig. 6B). The maximum likelihood tree using the Jukes-Cantor model showed that the two *Lens* cp genomes, *L. ervoides* and *L. culinaris*, were quite close to each other to a certain extent, at least for the examined proteins, whereas the other species in the group exhibited remarkable diversities. Interestingly, four *Trifolium* species accommodated larger diversity among each other in their cp genomes compared to the two lentils.

## Discussion

Legumes rank second among cereal crops in agricultural importance in terms of area harvested and total production^31^. The entire genome of the lentil was sequenced > 20 years ago^32^. Isolation of the cp DNA from a genome of ~ 4 Mbp^32^ in size is quite challenging. Recent developments in NGS technologies have enabled the investigation of genome organization and gene content in certain legume lineages, even using their cp.

This study concluded that the *L. ervoides* cp genome should be classified into IRLC species and determined that the cp genome has coexisting structural haplotypes alternative to each other. A typical cp genome size is 120 to 170 kb in land plants^33^ and generally contains 10 to 30 kb IR regions^34^. Usually, flip-flop recombination takes place between these IR regions. This recombination caused some plants to have coexisting structural haplotypes alternative to each other^18^. Although the cp genome structure was highly conserved, Kolodnert and Tewari (1979) revealed that pea (*Pisum sativum*) lost one copy of the IR copy regions^35^. Also, fava bean (*Vicia faba*), closely related to pea, contained a cp genome with a similar structure^36^. As the studies on the elucidation of the cp structure increased, a branch including IRLC species in the Papilionoideae subfamily of Fabaceae expanded^37^.

*L. ervoides*, with 122,722 bp in length, is analogous to *L. culinaris* and other legumes species, such as *C. arietinum*^38^, *Trifolium glanduliferum*^39^, and *V. faba*^31^. However, the number of genes in *L. ervoides* is similar to these three species; *C. arietinum* and *T. glanduliferum* species lack the *rps*16 gene as *L. ervoides*. In addition, the *inf*A gene is also absent in both *C. arietinum* and *L. ervoides*. The comparative gene content analysis of legume family members in this study showed great similarity, in agreement with previous reports.

The basic similarity between the two cp genomes of *Lens* species was investigated at the level of sequence differences^40^. These variant regions provide valuable insights for potential DNA barcode development to distinguish species. Regions of divergent hotspots between the two cp genome sequences in the coding and noncoding regions were determined and further examined using mVISTA analysis. In line with previous studies, in this study, noncoding regions were also more divergent as opposed to coding regions^41,42^. Overall, the divergence level between the two species was limited to a 0.25% difference, suggesting highly preserved cp genomes. Elevated amino acid substitution levels of certain genes, such as *ndh*B, *ndh*F, *rbc*L, *rpo*C2, and *ycf*2, point to the presence of an adaptive pressure most likely due to their functions. The effect of such changes requires further functional investigation. For species identification, *mat*K and *rbc*L are often suggested as molecular markers^43^. However, *mat*K carried only one amino acid difference between the two lentil species in this study, suggesting a limited potential for species distinction. In contrast, the *ycf*2 region with 8 nonsynonymous substitutions (13 nucleotide substitutions in total) can be an alternative marker for even close species within lentils. The potential of the *ycf2* region as a new identifier requires further genomic investigation, including other lentils and closely related species.

SSRs are commonly found in plant cp genomes and are widely used as molecular markers for population genetics and polymorphism studies^44^. In this study, *L. ervoides* and *L. culinaris* showed significantly similar repetitive sequence profiles as expected. SSR types and their frequencies for *L. ervoides* showed similar profiles to other previously reported species, especially closer to *V. faba* from the same family^31^.

Codon usage bias is also observed in plastome genes, and it plays a vital role in cp genome evolution as certain codon usage tendencies reshape cp genomes^45,46^. Various evolutionary mechanisms have affected the arrangement of codon usage bias^47^. The pattern of codon usage bias is specific to genes and varies among species. Such patterns are useful for population genetics and phylogenetic studies^48^. RSCU is used as a metric to measure codon usage bias^49^. The most encoded amino acid in *Phaseolus lunatus* belonging to the Fabaceae family is leucine. In contrast, tryptophan is the most encoded amino acid in *Coix lacryma-jobi* belonging to the Poaceae family^12,19^. *L. ervoides*, belonging to the Fabaceae family, encodes leucine the most frequently. This might suggest that the codon usage bias is not only specific to species but also carried through lineages, although additional representative genomes are required to draw a clear conclusion. Codon usage bias affects the efficiency of translation and protein folding^50,51^. Preferred codon usage can increase the translation of a gene by > 1000-fold^52^. In this analysis, *psb*N, *psa*I, *psb*I, *psb*E, *psb*K, *pet*D, and *ndh*C genes showed high frequencies of preferred codon usage. Thus, these genes can be predicted to have more expression as the function of MELP value and serve as potential targets in genetic engineering studies.

Conserved cp genomes are effective for determining interspecies phylogenetic relationships^53^. Complete cp sequencing studies using NGS technologies have revealed unknown phylogenetic relationships within the Plant Kingdom. In this study, the phylogenetic relationships of the *L. ervoides* plant have been revealed for the first time in the Plant Kingdom. The phylogenetic analysis on protein-coding genes showed that *L. culinaris*, *Vicia*, and *Trifolium* species were the closest to *L. ervoides*. In addition, the observed distance between *Vicia* species was dramatically larger compared to lentil species, most likely due to intraspecies divergence, as reported previously^54^. In addition, higher divergence was also observed among *Trifolium* species as opposed to lentils, and this divergence was previously explained by the variation in their repetitive sequence content^31,55,56^.

This study has provided deeper insights into a cp genomic structure from a wild lentil species. The results of this investigation will lead molecular genetic efforts to further advance agricultural practices. The existence of wild crop species should be more interrogated using modern molecular techniques to benefit from their long survival history.

## Materials and methods

### Plant materials, cp DNA isolation, and sequencing

*L. ervoides* was collected from the southeastern part of Turkey. The leaf samples of *L. ervoides* were gathered in compliance with national and international legislation and guidelines. It was registered by the herbarium of Akdeniz University Department of Field Crops, a validated voucher specimen was deposited with the voucher id of *L. ervoides*-01. *L. ervoides* species was identified botanically from Prof. Dr. Cengiz Toker, one of the co-author of the manuscript. It is not a nationwide protected plant. Thus, permission to collect the specimens was not required. Twenty grams of freshly harvested leaves were stored immediately at 4 °C for 3 days to reduce their starch accumulation before long-term storage in liquid nitrogen. The quality of the isolates was determined after cp DNA isolation by an agarose gel electrophoresis following the protocol of Shi et al.^57^. At least 1 μg input DNA was sent to the Beijing Genome Institute (Hong Kong, China) for DNA quantification and sequencing with NanoDrop (ND-1000; Thermo Co.) and DNA Nanoball (DNB) method with the BGISEQ-500 platform. The fragmented genomic DNA has been size-selected for sequencing library preparation to have a 200 to 400 bp range and purified using XP-AMPure magnetic beads (Agencourt). Then, 3′-end adenylation and heat denaturation were followed by a library creation step through splint oligo sequences and single-stranded circular DNA (ssCir DNA). With the rolling-circle replication method, DNA containing > 300 copies-DNA in ssCir DNAs was turned into a DNB. Using high-density DNA nanochip technology, DNBs were transferred to nanoarrays in specific patterns. Then, 150 bp paired-end reads were obtained with combinatorial Probe-Anchor Synthesis and stored in a fastq file format.

### cp genome assembly and annotation

High-quality and adapter-spliced raw sequencing reads were used for the de novo genome assembly pipeline designed specifically for plastid chromosomes using GetOrganelle^58^. This pipeline essentially utilized the Spades de novo assembly tool^59^ with the parameter settings “-t 4 -F embplant_pt -R 15 -w 102,” which sets the “word size” of 102 with 4 computed threads through 15 rounds. The initial run was fed with the existing *L. culinaris* cp DNA (NC_027152.1) as the seed sequence, and the assembled contigs were searched against a precompiled plant plastid genome database “embplant_pt.” All possible scaffolds generated by the assembler were visually investigated using the Bandage software^60^ and manually curated for contig orientations based on *L. culinaris* cp DNA (with custom scripts). The raw sequencing reads were aligned back to the drafted assembly sequence using bowtie2 with its default parameters^61^. The read support of each variant position was inspected manually.

The GeSeq^62^ online tool, which internally searches with various approaches, such as ARAGORN, Chloe, HMMER, tRNAscan-SE, blatN, and BlatX, was used for the initial annotation of loci for both coding and noncoding regions in the *L. ervoides* cp DNA. Additionally, gene coordinates from the *L. culinaris* cp genome through pairwise sequence alignment were compared to ensure the integrity of gene annotations. Organellar Genome DRAW (OGDRAW)^63^ was used to visualize the structural organization of the genome and gene annotations.

### Comparative genome and divergence analysis

MUMmer was used for binary sequence analysis (dot-graph) between *L. ervoides* cp genome scaffolds and *L. culinaris*^64^. For further comparative genomic investigation of similarity blocks, MAUVE Alignment software^65^ and mVISTA software with Shuffle-LAGAN mode were utilized^66,67^.

The divergent regions between cp genomes of *L. ervoides* and *L. culinaris* were determined using their pairwise genome alignment, and repetitive sequence regions discovered by miropeats were masked^68^. Sequence disagreements (mismatches and indels) between the two species with a 1000 bp (200 bp sliding) window were calculated using custom python scripts for genome-wide divergent hot spots. Synonymous and nonsynonymous substitutions (depending on coding status) were determined for genic regions using the canonical amino acid codon table.

### Analysis of repetitive sequences and codon usage bias

Forward, reverse, and complementary sequences with palindromic repeats were identified using the online tool REPuter^69^ (minimum repeat = 30 bp, hamming distance = 3). The Tandem Repeats Finder online tool was utilized to identify tandem repeats^70^. Nucleotide repeat sequences were determined using the MIcroSAtellite identification tool^71^.

Codon usage statistics were calculated by functions from an R package called “cordon” (version 1.1.3)^72^ using only protein-coding regions. These statistics include MILC and MELP and the basic codon usage frequencies^30^. RSCU values were computed based on the definition as the ratio of the observed frequency of codons to the expected uniform codon usage.

### Phylogenetic analysis

MAFFT^73^ was used for multiple sequence alignment, and aligned sequences were visually inspected using Seaview^74^ software. For phylogenetic analysis of whole cp genomes of several species, a maximum likelihood tree was constructed with the Jukes-Cantor^75^ model, allowing < 10% missing data for positions not covered or with ambiguous bases. All calculations and drawings were completed using MEGA11 software^76^ with 100 bootstraps. *A. thaliana* and *Z. mays* were chosen as the outgroups. Several existing cp genomes of Papilionoideae were obtained from the National Center for Biotechnology Information nucleotide database. These genomes were *Astragalus nakaianus* (NC_028171.1), *C. arietinum* (NC_011163.1), *L. culinaris* (NC_027152.1), *Lathyrus sativus* (NC_014063.1), *Medicago truncatula* (NC_003119.8), *P. sativum* (NC_014057.1), *Trifolium aureum* (NC_024035.1), *Trifolium boissieri* (NC_025743.1), *T. glanduliferum* (NC_025744.1), *Trifolium strictum* (NC_025745.1), *Vicia sativa* (NC_027155.1), *V. faba* (KF042344.1), *Wisteria floribunda* (NC_027677.1), and, as outgroups, *Z. mays* (NC_001666.2) and *A. thaliana* (NC_000932.1). Sequences of shared protein-coding genes were concatenated for the phylogenetic investigation of more divergent members of Papilionoideae.

## Supplementary Information

Supplementary Information.

## Data Availability

The raw sequencing datasets generated during the current study are available in the ENA (European Nucleotide Archive) of EMBL-EBI under the accession number PRJEB47534. The assembled whole cp genome sequence of *L. ervoides* can be accessed through sample identification number ERS7635408.
