# Supplementary material for: Complete chloroplast genome sequence of Lens ervoides and comparison to Lens culinaris
Source: Sci Rep. 2022 Sep 5;12:15068. doi: 10.1038/s41598-022-17877-7 (PMC9445179; doi:10.1038/s41598-022-17877-7)
Supplement: Supplementary file 1 — Supplementary Information. [file 41598_2022_17877_MOESM1_ESM.docx]

**Supplemental Material**

**Supplemental Table 1.** Repetitive sequence positions within the *L. culinaris* cp genome that are discovered by miropeats (v2.02).

| ***Species*** | **Start** | **End** |
| --- | --- | --- |
| *Lens_culinaris* | 19022 | 19186 |
| *Lens_culinaris* | 21246 | 21410 |
| *Lens_culinaris* | 54483 | 54684 |
| *Lens_culinaris* | 54655 | 54856 |
| *Lens_culinaris* | 54860 | 55035 |
| *Lens_culinaris* | 54956 | 55102 |
| *Lens_culinaris* | 55100 | 55300 |
| *Lens_culinaris* | 68767 | 68991 |
| *Lens_culinaris* | 84153 | 84261 |
| *Lens_culinaris* | 84233 | 84341 |
| *Lens_culinaris* | 103588 | 103812 |

**Supplemental Table 2.** Substitution rates in the protein coding genes of *L. ervoides* with respect to *L. culinaris*. Total number of detected synonymous or non-synonymous changes and the putative amino acid substitutions are given based on the residue positions on the protein. A gap (‘-’) represents a deletion (in the *L. ervoides* genome).

| **#Gene** | **Number of total variants detected** | **Number of Non-synonymous variants** | **Number of Synonymous variants** | **Nonsynonymous changes on the protein** | **Description** |
| --- | --- | --- | --- | --- | --- |
| **accD** | 2 | 2 | 0 | K62Q, L156F | Acetyl-CoA carboxylase carboxyltransferase beta subunit |
| **atpA** | 1 | 0 | 1 |  | ATP synthase CF1 alpha subunit |
| **atpF** | 1 | 0 | 1 |  | ATP synthase CF0 subunit I |
| **ccsA** | 1 | 0 | 1 |  | Cytochrome c heme attachment protein |
| **cemA** | 1 | 0 | 1 |  | Chloroplast envelope membrane protein |
| **matK** | 1 | 1 | 0 | N192K | Maturase K |
| **ndhA** | 1 | 0 | 1 |  | NdhA |
| **ndhB** | 4 | 3 | 1 | F413L, Q442E, L484V | NADH-plastoquinone oxidoreductase subunit 2 |
| **ndhF** | 4 | 4 | 0 | A538P, **TFFV560-**, H576D, Y631S | NADH-plastoquinone oxidoreductase subunit 5 |
| **ndhH** | 1 | 1 | 0 | I249V | NADH-plastoquinone oxidoreductase subunit 7 |
| **psaB** | 1 | 0 | 1 |  | Photosystem I P700 apoprotein A2 |
| **psbD** | 1 | 0 | 1 |  | Photosystem II protein D2 |
| **rbcL** | 3 | 3 | 0 | P50A, D94E, G449A | Ribulose-1,5-bisphosphate carboxylase/oxygenase large subunit |
| **rpl16** | 1 | 0 | 1 |  | Ribosomal protein L16 |
| **rpl2** | 1 | 1 | 0 | E132G | Ribosomal protein L2 |
| **rpl20** | 1 | 1 | 0 | S30N | Ribosomal protein L20 |
| **rpl23** | 1 | 1 | 0 | R63Q | Ribosomal protein L23 |
| **rpl33** | 1 | 0 | 1 |  | Ribosomal protein L33 |
| **rpoA** | 1 | 1 | 0 | K330N | RNA polymerase alpha subunit |
| **rpoB** | 4 | 2 | 2 | M609I, M728L | RNA polymerase beta subunit |
| **rpoC1** | 1 | 0 | 1 |  | RNA polymerase beta |
| **rpoC2** | 5 | 3 | 2 | G936C, Y1021S, Q1094E | RNA polymerase beta' subunit |
| **rps11** | 2 | 0 | 2 |  | Ribosomal protein S11 |
| **rps14** | 1 | 1 | 0 | E42Q | Ribosomal protein S14 |
| **rps2** | 2 | 1 | 1 | K136R | Ribosomal protein S2 |
| **rps3** | 1 | 1 | 0 | F42C | Ribosomal protein S3 |
| **rps4** | 2 | 0 | 2 |  | Ribosomal protein S4 |
| **rps7** | 2 | 2 | 0 | L11I, Y60S | Ribosomal protein S7 |
| **rps8** | 1 | 1 | 0 | L40I | Ribosomal protein S8 |
| **ycf2** | 13 | 8 | 5 | D829E, L988I, T1035S, K1246M, M1290R, F1436L, Q1717E, I1751M | Hypothetical chloroplast RF21 |

**Supplemental Table 3.** Substitution rates in the non-coding genes of *L. ervoides* with respect to *Lens culinaris*. A gap (‘-’) represents an insertion (in the *L. ervoides* genome).

| **#Gene** | **Number of total variants detected** | **Changes in respect to transcription start site in the gene.** |
| --- | --- | --- |
| trnQ-UUG | 1 | A113T |
| rrn23 | 5 | C289A, -1256TAACCACGCGAGCAGGGTAGGACGAAAAGAT, G1362C, G1363A, G1568T |

**Supplemental Table 4.** The codon recognition pattern, codon usage for *L. ervoides* and amino acid occurrence in the coding genes of *L. ervoides* cp genome. CU: codon usage.

| **Codon** | **Amino Acid** | **CU *L. ervoides*** | **RSCU *L. ervoides*** | **Total occurrences** |
| --- | --- | --- | --- | --- |
| TAA | * | 47 | 1.78 | 79 |
| TAG | * | 13 | 0.49 |  |
| TGA | * | 19 | 0.72 |  |
| GCT | A | 541 | 1.84 | 1171 |
| GCC | A | 167 | 0.57 |  |
| GCA | A | 334 | 1.14 |  |
| GCG | A | 129 | 0.44 |  |
| TGT | C | 173 | 1.55 | 222 |
| TGC | C | 49 | 0.44 |  |
| GAT | D | 600 | 1.62 | 739 |
| GAC | D | 139 | 0.37 |  |
| GAA | E | 759 | 1.52 | 997 |
| GAG | E | 238 | 0.47 |  |
| TTT | F | 842 | 1.44 | 1167 |
| TTC | F | 325 | 0.55 |  |
| GGT | G | 504 | 1.44 | 1392 |
| GGC | G | 141 | 0.40 |  |
| GGA | G | 547 | 1.57 |  |
| GGG | G | 200 | 0.57 |  |
| CAT | H | 346 | 1.55 | 446 |
| CAC | H | 100 | 0.44 |  |
| ATT | I | 914 | 1.54 | 1774 |
| ATC | I | 288 | 0.48 |  |
| ATA | I | 572 | 0.96 |  |
| AAA | K | 778 | 1.56 | 996 |
| AAG | K | 218 | 0.43 |  |
| TTA | L | 735 | 2.09 | 2108 |
| TTG | L | 415 | 1.18 |  |
| CTT | L | 443 | 1.26 |  |
| CTC | L | 102 | 0.29 |  |
| CTA | L | 283 | 0.80 |  |
| CTG | L | 130 | 0.37 |  |
| ATG | M | 452 | 1.0 | 452 |
| AAT | N | 687 | 1.57 | 875 |
| AAC | N | 188 | 0.42 |  |
| CCT | P | 335 | 1.64 | 815 |
| CCC | P | 149 | 0.73 |  |
| CCA | P | 248 | 1.21 |  |
| CCG | P | 83 | 0.40 |  |
| CAA | Q | 563 | 1.59 | 704 |
| CAG | Q | 141 | 0.40 |  |
| CGT | R | 252 | 1.40 | 1076 |
| CGC | R | 73 | 0.40 |  |
| CGA | R | 232 | 1.29 |  |
| CGG | R | 87 | 0.48 |  |
| AGA | R | 325 | 1.81 |  |
| AGG | R | 107 | 0.59 |  |
| TCT | S | 438 | 1.85 | 1420 |
| TCC | S | 215 | 0.90 |  |
| TCA | S | 286 | 1.20 |  |
| TCG | S | 110 | 0.46 |  |
| AGT | S | 295 | 1.24 |  |
| AGC | S | 76 | 0.32 |  |
| ACT | T | 419 | 1.66 | 1007 |
| ACC | T | 172 | 0.68 |  |
| ACA | T | 317 | 1.25 |  |
| ACG | T | 99 | 0.39 |  |
| GTT | V | 424 | 1.54 | 1101 |
| GTC | V | 129 | 0.46 |  |
| GTA | V | 414 | 1.50 |  |
| GTG | V | 134 | 0.48 |  |
| TGG | W | 341 | 1.0 | 341 |
| TAT | Y | 584 | 1.66 | 703 |
| TAC | Y | 119 | 0.33 |  |

**A**


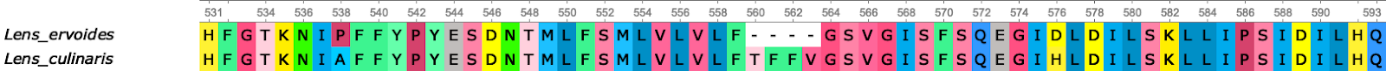


**B**


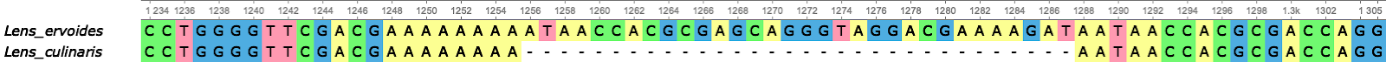


**Supplemental Figure 1. A)** The deletion in the *ndh*F protein sequence of *L.. ervoides* resulting in a loss of 4 amino acids in frame at the position of 560. **B)** The 31-nucleotide insertion in the *L. ervoides* 23S ribosomal RNA transcript region.
